# Post-translational S-glutathionylation of cofilin increases actin cycling during cocaine seeking

**DOI:** 10.1371/journal.pone.0223037

**Published:** 2019-09-24

**Authors:** Anna Kruyer, Lauren E. Ball, Danyelle M. Townsend, Peter W. Kalivas, Joachim D. Uys

**Affiliations:** 1 Department of Neuroscience, Medical University of South Carolina, Charleston, SC, United States of America; 2 Department of Cell and Molecular Pharmacology & Experimental Therapeutics, Medical University of South Carolina, Charleston, SC, United States of America; 3 Department of Drug Discovery and Pharmaceutical Sciences, Medical University of South Carolina, Charleston, SC, United States of America; Sao Paulo Federal University, BRAZIL

## Abstract

Neuronal defense against oxidative damage is mediated primarily by the glutathione redox system. Traditionally considered a mechanism to protect proteins from irreversible oxidation, mounting evidence supports a role for protein S-glutathionylation in cell signaling in response to changes in intracellular redox status. Here we determined the specific sites on the actin binding protein cofilin that undergo S-glutathionylation. In addition, we show that S-glutathionylation of cofilin reduces its capacity to depolymerize F-actin. We further describe an assay to determine the S-glutathionylation of target proteins in brain tissue from behaving rodents. Using this technique, we show that cofilin in the rat nucleus accumbens undergoes S-glutathionylation during 15-minutes of cued cocaine seeking in the absence of cocaine. Our findings demonstrate that cofilin S-glutathionylation is increased in response to cocaine-associated cues and that increased cofilin S-glutathionylation reduces cofilin-dependent depolymerization of F-actin. Thus, S-glutathionylation of cofilin may serve to regulate actin cycling in response to drug-conditioned cues.

## Introduction

Protein S-glutathionylation is the reversible covalent linkage of glutathione (GSH) to cysteine residues on target proteins. Approximately 200,000 cysteines are encoded in the mammalian genome, 10–20% of which are redox sensitive under biological conditions [1] and there is a correlation between organism cysteine content and biological complexity [2]. Given its intracellular abundance (1–10 mM) and high electron donating capacity, GSH is considered the major low molecular weight antioxidant in mammalian cells [3, 4]. During oxidative stress, protein S-glutathionylation may serve as a depot for GSH, since oxidized glutathione (GSSG) is quickly removed from the cell [5]. S-glutathionylated sulfhydryl groups are protected from irreversible oxidation to sulfinic or sulfonic acid, the latter of which is largely resistant to cellular repair and results in proteosomal degradation [6]. Furthermore, S-glutathionylation can induce conformational changes, sterically block sites of catalysis or posttranslational modification, and confers a negative charge to proteins, all actions that can affect protein function [7, 8]. However, not all cysteines are able to undergo S-glutathionylation, as a low pKa local environment is required to make the sulfhydryl groups susceptible to modification [9]. Although other post-translational modifications such as phosphorylation and ubiquitinylation have been well described, S-glutathionylation as a regulatory modification with reversible effects on protein function is a relatively recent conceptual advance [6].

Actin polymerization is an important feature of many essential cellular activities, including motility and synaptic plasticity. In the brain, rapid morphological changes in dendritic spines depend on actin dynamics, and shifts from monomeric G-actin to polymeric F-actin drive new spine formation, spine expansion, and synaptic plasticity [10, 11]. Recent studies demonstrate that proteins involved in actin cycling are highly sensitive to oxidative stress, with actin itself being the target of S-glutathionylation [12], and treatment with hydrogen peroxide drives cytoskeletal rearrangements and filamentous actin (F-actin) fragmentation *in vitro* [13].

The actin cofactor cofilin binds to actin filaments, promoting F-actin branching, inducing filament severing, and facilitating removal of monomeric actin [14, 15]. Phosphorylation of cofilin (Ser3, p-cofilin) impairs its binding to and severing of F-actin [16], while cofilin de-phosphorylation promotes actin binding and branching and dendritic spine remodeling [17]. Cofilin has also been identified as a target of post-translational S-glutathionylation [18], but the effect of this modification on the function of cofilin in modulating actin dynamics has not been tested. For this reason, we induced S-glutathionylation of cofilin *in vitro* to determine which of its four cysteines are potential targets of glutathionylation and whether this modification can alter its depolymerizing effect on F-actin. Since transient dendritic spine head expansion can be induced in accumbens neurons by cocaine-conditioned environmental cues [19], we also examined whether cocaine cues are associated with altered S-glutathionylation of cofilin.

## Methods

### Cocaine self-administration

All animal procedures were approved by the Institutional Animal Care and Use Committee at the Medical University of South Carolina. Adult (250-300g) male Sprague Dawley rats (Charles River Laboratories) were housed in a vivarium on a reverse 12h/12h light/dark cycle with access to food and water *ad libitum*. Rats were trained to self-administer cocaine using surgical and behavioral techniques described previously [20]. Briefly, animals were anesthetized with ketamine/xylazine (100 mg/kg and 7 mg/kg, IM), implanted with jugular catheters and allowed to recover from surgery for 1 week. After recovery, rats underwent 2h of food training to learn the operant task. During food training, animals received one food pellet in the absence of cues per active lever press. The following day, animals began daily 2-hour self-administration sessions, during which, presses on an active lever yielded IV cocaine delivery (0.2 mg/infusion per active lever press followed by a 20s timeout). Each active lever press and cocaine infusion was paired with tone and light cues for 5s. After 10 days of self-administration, rats were placed in the same boxes, but lever presses had no consequence. Following 10 days of extinction training, rats in the extinction group as well as yoked saline controls were sacrificed 24h after the last session. Rats undergoing reinstatement were placed in the self-administration boxes a final time and tone-light pairing were restored to the active lever, but active lever presses yielded no cocaine delivery. Reinstated rats were sacrificed after 15-minutes in the self-administration chamber. Control yoked saline rats underwent the same treatments and training as self-administering rats, but received passive infusions of saline paired with tone and light cues.

### S-glutathionylation switch assay and Western blotting

A variation on the biotin switch protocol [21] was used to determine levels of cofilin S-glutathionylation in the nucleus accumbens in rats following cocaine self-administration and extinction. Sacrifice was conducted by live decapitation and the nucleus accumbens from both hemispheres was dissected on ice [22]. Tissue proteins were homogenized in buffer containing N-ethylmaleimide (Sigma-Aldrich), which binds non-glutathionylated thiols [23]. After protein extraction, an aliquot of total protein from each lysate was stored at -20C for later use. Native S-glutathionylated cysteines were de-glutathionylated via reaction with glutaredoxin-1 (Cayman Chemical) and the previously S-glutathionylated cysteines from 600 μg protein were bound to thiol resin (Sigma-Aldrich) prior to elution in 20mM DTT and analysis by Western blot. Eluted samples were run alongside total protein samples from the same rat and abundance of natively S-glutathionylated cofilin was quantified as well as quantity of cofilin in 15 μg of the original lysate. Primary antibody against cofilin (Abcam, ab42824) was diluted in Odyssey blocking buffer (1:1000). Blots were imaged using the LI-COR Odyssey system and bands were quantified using FIJI according to NIH guidelines for analyzing electrophoretic gels (https://imagej.nih.gov).

### Pyrene F-actin depolymerization assay

Cofilin (Cytoskeleton Inc.) was resuspended in H_2_O (5mg/mL) and passed through Bio-spin 6 Tris columns (Bio-Rad) according to the manufacturer’s instructions for buffer exchange to 10mM phosphate buffer pH 7.4. A portion of the cofilin was incubated in 10mM GSH and 10 μM disulfiram for 30 min at 37C to induce S-glutathionylation. Proteins were then passed through bio-spin filter columns to remove excess glutathionylation reagents. Non-muscle actin (3 mg) and pyrene-labeled muscle actin (Cytoskeleton Inc.) were each resuspended in 150 μl H_2_O supplemented with 0.2 mM ATP and combined in a 1:1 ratio to produce an actin stock with 0.02 mg/mL final concentration of each protein. General actin buffer (supplemented with 4.8 μl ATP, 4.8 mL final volume) was combined with 240 μl of actin stock and 120 μl of actin polymerization buffer in a light-protected tube. This diluted actin stock solution was gently mixed by pipetting up and down and left at room temperature for 1 hour. General actin buffer (9.6 mL, pH 8.0) and 2 mL of diluted F-actin stock were added to cuvettes and combined with either S-glutathionylated or native cofilin (final concentration 20 μg/mL). Pyrene-labeled F-actin fluorescent absorbance readings were recorded at 344 nm/475 nm for 12 min.

### Tryptophan fluorescence spectroscopy

Aliquots from the cofilin and cofilin-SSG preparations that were used for the F-actin depolymerization assay, including cofilin treated with disulfiram only, were used to measure intrinsic fluorescence by tryptophan fluorescence spectroscopy as previously described [24]. Protein tryptophan fluorescence was recorded on an F 2500 spectrofluorometer (Hitachi) using 10 × 10 × 40 mm quartz cuvettes. Excitation and emission slits were 2.5 and 5.0 nm, respectively. The excitation wavelength was 295 nm to minimize an effect of protein tyrosines and phenylalanines. Background spectra were subtracted from final emission of the protein.

### Mass spectrometry

The LC-MS/MS analysis of cofilin-SSG was similar to our previously published method [25, 26]. S-glutathionylation of recombinant human cofilin 1 (P23528) (Cytoskeleton Inc.) was induced by incubation with 10 μM tetraethylthiuram disulfide (disulfiram, Sigma) in the presence of reduced L-glutathione (Sigma) for 30 minutes at 37°C. The sample was loaded onto a 4–12% Bis-Tris non-denaturing precast gel and separated by electrophoresis using MOPS buffer and a Criterion Cell system (Bio-Rad). Following overnight staining with Proto Blue Safe (National Diagnostics), the protein band was destained, and digested overnight at 37° C with Lys-C (Roche). The peptides were extracted by repeated incubation with a 5% formic acid (FA) in 50% acetonitrile (ACN) solution. The extracted peptides were lyophilized in a Speed Vac (Thermo Scientific) and redissolved in a 0.2% FA solution. The peptides were loaded on a trap column, eluted, and separated on a 75 μm x 15 cm fused-silica column packed in house with C18 reverse-phase resin (YMC-ODS-AQ; 5-μm particles; 120-Å pore; Waters Corp). The gradient was from 5–50% in 120 min. Both solvent A (2% ACN) and B (98% ACN) contained 0.2% FA. A U3000 nano-LC system (LC Packings) was used at a flow rate of 200 nL/min. Peptides were mass analyzed by a Thermo Scientific Orbitrap Elite mass spectrometer capable of collision induced dissociation (CID), higher-energy collisional dissociation (HCD), and electron transfer dissociation (ETD) fragmentation. Given the complex fragmentation of S-glutathionylated peptides by collisional dissociation, which can impede detection by automated database searching algorithms, multiple LC-MS/MS analyses were performed to determine the optimal fragmentation mode to directly identify S-glutathionylated peptides including CID, HCD, ETD, ETD with supplemental activation, and the acquisition of ETD spectra following neutral loss of 129 Th from precursor ions fragmented by CID or HCD. Mass spectra were acquired in data-dependent mode with one FTMS survey scan with a mass range of m/z 400–1700 acquired in the Orbitrap followed by tandem mass spectra (MS/MS) of the 7 most intense precursor ions. Product ions of HCD were detected in the Orbitrap. CID and ETD product ions were detected in the ion trap. The automatic gain control target value in the Orbitrap was 10^6^ for the survey MS scan at a resolution of 60,000 at m/z 400. Fragmentation in the ion trap was performed with a target value of 1000 ions. The threshold count was 500 for CID and ETD. The default ETD reaction times for a given charge state were utilized. Dynamic exclusion was enabled with a repeat count of 3, repeat duration of 30 sec, exclusion list of 50, and exclusion duration of 100 sec. Glutathione increases the mass of cysteine by 305.0682 Da and during CID fragmentation undergoes the neutral loss of γ glutamic acid (-129.04 Da). By ETD, the disulfide bond between the cysteine and the glutathione moiety is preferentially broken resulting in the loss of glutathione from the precursor and fragment ions generating the diagnostic glutathione ion at m/z 308. The CID, HCD, and ETD MS/MS spectra were searched using MASCOT (Version 2.4.01) and SEQUEST HT search algorithms utilizing Proteome Discoverer 1.4 (Thermo Scientific). For both algorithms, the search parameters allowed for 2 missed cleavages, precursor mass tolerances of ± 20 ppm, and fragment mass tolerances of ± 0.8 Da. Additional searches using default parameters were performed with MaxQuant [27] and Protein Prospector [28]. Dynamic modification of cysteine included S-glutathionylation (+305.0682 Da). Algorithms were also programmed to search ETD spectra for the presence of the diagnostic ion at m/z 308 and CID spectra for neutral loss of 129 Da. All searches were against a database containing the amino acid sequence for cofilin 1 and the digestion enzyme. All identified and reported spectra were manually inspected. LC-MS/MS with alternating CID/ETD fragmentation followed by MaxQuant and Protein Prospector database searches with diagnostic fragmentation patterns yielded the most true-positive identifications.

### MS analysis of immunoprecipitated cofilin

Cofilin was immunoprecipitated from 5 and 10 mg aliquots of nucleus accumbens protein and eluted in acidic solution. The pH was adjusted to 7.5 with 1 M ammonium bicarbonate and Tris buffer and the protein digested overnight with LysC (Waco). Peptides were desalted using C18 ZipTips (Millipore) and analyzed by LC-MS/MS using a 30 cm x 75 μm C18 reverse phase resin packed analytical column (Dr. Maisch; 1.9 μm particle size) in-line with an Orbitrap Elite. For the most sensitive mode of instrument operation, peptides were fragmented by CID and product ions were detected in the ion trap. For targeted detection of S-glutathionylated peptides, the instrument was programmed to first acquire MS/MS data on precursor ions with mass to charge ratios corresponding to previously detected *in vitro* modified peptides of cofilin (see S1 Table, S1 Fig). It should be noted that human and rat cofilin 1 sequences are identical. Raw data were searched using database searching algorithms as described above. Parameters included variable modification of cys with glutathione, nitrosylation, oxidation (16, 32, 48 Da) or N-ethylmaleimide; phosphorylation of ser, thr, tyr; and lys acetylation. Database hits of modified peptides were manually verified and the raw data were manually interrogated for S-glutathionylated peptides.

### Statistical analysis

Numerical data were analyzed using GraphPad Prism 7 and are presented as mean ± standard error of the mean. Statistical significance was determined using either a Student’s t test, nested one-way ANOVA with Sidak’s multiple comparison test, one-way ANOVA with Tukey’s post-hoc test, or a two-way ANOVA. In all cases, *p*-values < 0.05 were considered significant.

## Results

### LC-MS/MS confirms cofilin S-glutathionylation on C39, C80, C139, and C147

**Fig 1:**
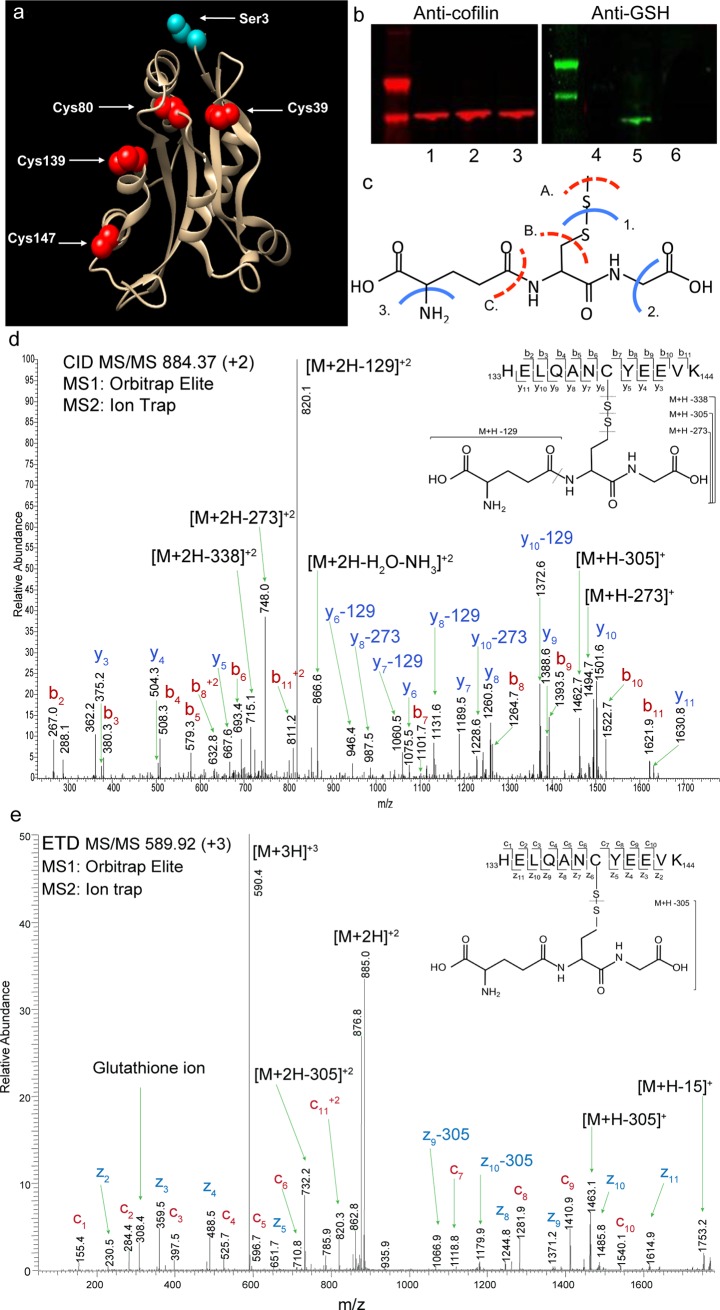
Mass spectra confirm S-glutathionylation of cofilin. (a) 3D representation of cysteine residues (red) of cofilin that serve as targets of S-glutathionylation, and the phosphorylation site at serine 3 (blue). (b) Double near-infrared Western blot confirmation of *in vitro* S-glutathionylation of recombinant cofilin. Anti-cofilin blot showing recombinant cofilin (1), cofilin + disulfiram + GSH (2), cofilin + disulfiram (3). Anti-GSH blot showing recombinant cofilin (4), cofilin + disulfiram + GSH (cofilin-SSG) (5) and cofilin + disulfiram (6). (c-e) Cofilin was S-glutathionylated *in vitro* using tetraethylthiuram disulfide, digested with LysC, and the resulting peptides were analyzed by LC-MS/MS with alternating (d) CID and (e) ETD fragmentation modes. (d) Collision-induced dissociation tandem mass spectrum (CID MS/MS) of S-glutathionylated cys139. CID results in fragmentation of the peptide backbone between the amino acids yielding b’ and y’ type ions. Collisional dissociation of glutathionylated cofilin 133–144 resulted in a complex fragmentation pattern with the most intense product ions resulting from fragmentation of glutathione. Glutathione, which increases the mass of cysteine by 305.07 Da, may undergo fragmentation resulting in partial neutral losses of 129 Da (γ-glutamic acid), 305 Da, 273 Da, and 338 Da from b, y, and precursor ions (labile bonds of glutathione indicated by dashed lines in (c)). (e) Electron transfer dissociation tandem mass spectrum (ETD MS/MS) of S-glutathionylated cys139. ETD results in fragmentation of the peptide backbone between the amino group and the alpha carbon yielding c’ and z’ type ions. By ETD, the disulfide bond is preferentially fragmented resulting in the loss of glutathione (M+H-305) and the generation of a diagnostic glutathione ion at m/z 308.4. See supplemental data for spectra of other S-glutathionylated cysteines within cofilin.

Three-dimensional modeling of cofilin illustrates all four cysteine residues that serve as potential sites for S-glutathionylation, as well as the most widely reported site for serine phosphorylation by LIM kinase (Fig 1A). Additional phosphorylation sites on cofilin, not shown here, include threonine 63, tyrosine 82, and serine 108 [29]. We incubated recombinant cofilin with disulfiram and GSH *in vitro* to induce its S-glutathionylation (Fig 1B). Electron transfer (ETD, Fig 1E, S1A–S1D Fig) and collision-induced dissociation (CID, Fig 1D, S1A–S1D Fig) tandem mass spectrometry revealed S-glutathionylation at all four of its cysteine residues (C39, C80, C139, and C147). Current automated database searching does not allow high confidence identification of S-glutathionylated cysteine residues. Therefore, we optimized parameters and mass spectrometry fragmentation modes, thereby enabling direct detection of S-glutathionylated peptides (S1 and S2 Tables and S1 Fig).

### Cofilin-SSG has altered 3D conformation

**Fig 2:**
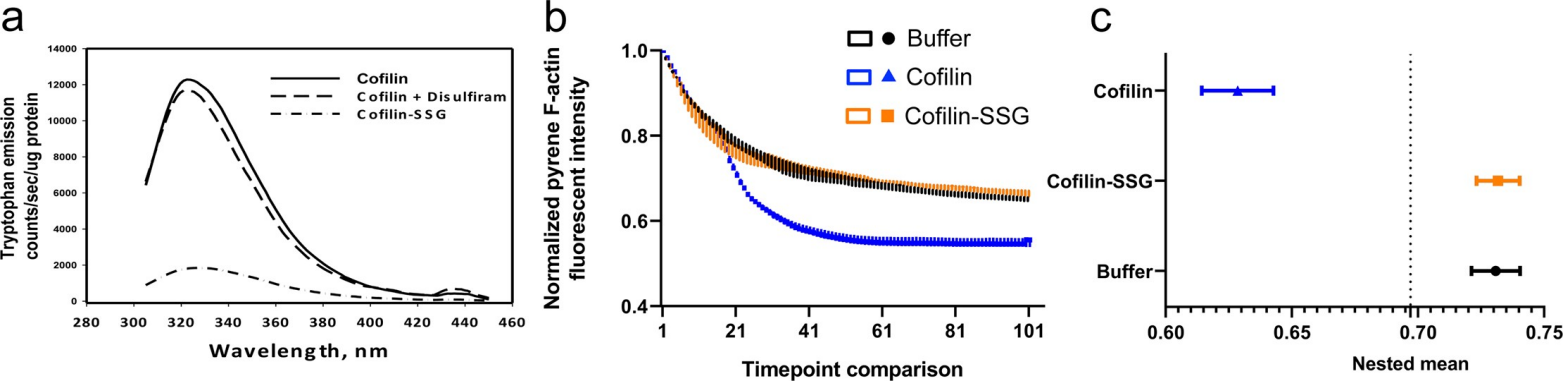
Cofilin-SSG has an altered 3D conformation and is inhibited in its ability to de-polymerize F-actin. (a) Intrinsic fluorescent analysis of cofilin-SSG shows a change in the 3D structure. Recombinant cofilin was S-glutathionylated with disulfiram and GSH (cofilin-SSG) and showed a large decrease in tryptophan fluorescence, indicating a 3D structural change. Non-modified cofilin and cofilin + disulfiram only (no GSH) showed no change in intrinsic fluorescence. (b, c) F-actin depolymerization was inhibited by S-glutathionylated cofilin. Pyrene F-actin was incubated with either cofilin or cofilin-SSG at pH 8.0. Cofilin depolymerized F-actin as expected, as indicated by a decrease in pyrene fluorescence, but cofilin-SSG showed no de-polymerization activity. Buffer only (Tris-HCl, pH 8.0) served as a negative control. (F _(2, 906)_ = 111.1, p < 0.001; p = 0.001, buffer vs. cofilin and p = 0.99, buffer vs. cofilin-SSG) by nested one-way ANOVA followed by Sidak’s multiple comparisons test. Data is presented as mean ± s.e.m. in b, c; n = 3/grp.

Post-translational modification of any of the cysteine residues on cofilin is positioned to potentially alter the 3D structure of the protein, but would not appear to directly interfere with serine 3 where phosphorylation of cofilin markedly inhibits actin depolymerization (Fig 1A) [30]. Using fluorescence spectroscopy, we found that intrinsic fluorescence of cofilin-SSG was reduced relative to non-modified cofilin or cofilin incubated with disulfiram alone. The hypochromic shift observed in the cofilin-SSG spectrum can be attributed to polar shielding of tryptophan residues proximal to S-glutathionylation sites, indicating that an S-glutathionylation dependent altered tertiary conformation was produced (Fig 2A).

### Cofilin-SSG is inhibited in its ability to de-polymerize F-actin

Next the capacity of cofilin and cofilin-SSG to depolymerize F-actin was determined using an *in vitro* activity assay. We incubated pyrene-labeled actin, which produces enhanced fluorescence in the filamentous state compared to the non-polymerized G-actin form, with cofilin-SSG or non-modified cofilin. Incubation with cofilin-SSG increased fluorescence emission compared to non-modified cofilin (Fig 2B and 2C), indicating that non-modified cofilin caused a rapid de-polymerization of F-actin into its monomeric G form and that this process was impaired in the presence of cofilin-SSG. Thus, S-glutathionylation of the cysteine residues of cofilin produces similar effects on actin stability *in vitro* as p-cofilin [31], prolonging F-actin stability. Taken together, our findings suggest that S-glutathionylation of cofilin induces a change in 3D conformation, which may contribute to an altered capacity to bind to and de-polymerize F-actin to its monomeric form. The surface exposure of the cysteines in cofilin may make them effective redox sensors, enabling rapid conformational shifts in response to oxidative stressors, with possible effects on actin cycling and cell morphology.

### Cofilin S-glutathionylation is increased in response to cocaine-associated cues

**Fig 3:**
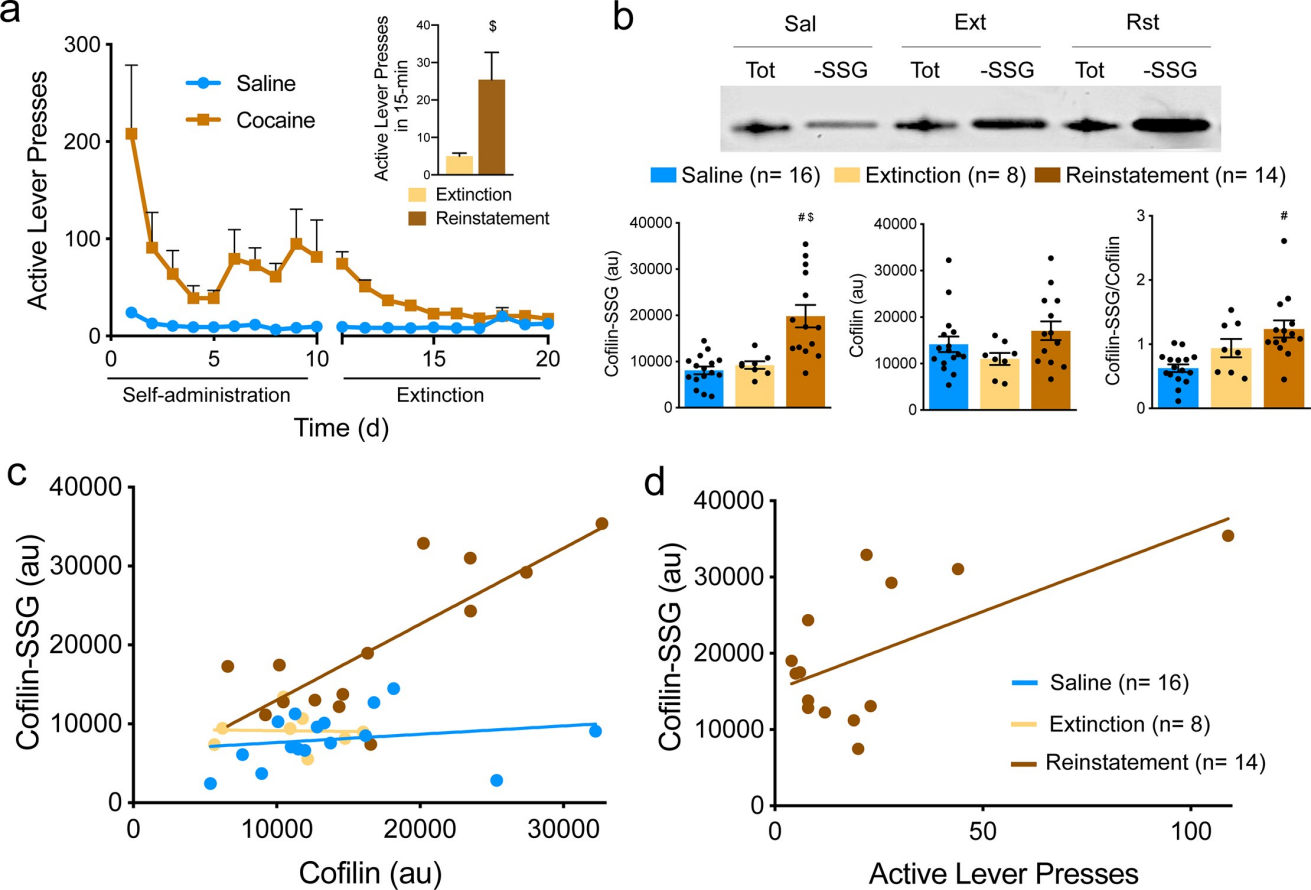
Cofilin S-glutathionylation is increased during 15-minutes of cue-induced cocaine seeking in the absence of cocaine. (a) Active-lever presses during cocaine self-administration and extinction training (n = 16, Saline; n = 22, Cocaine; effect of treatment p < 0.0001 by 2-way ANOVA, F _(19, 709)_ = 51.85). 15-min of cue exposure elevated lever pressing relative to the first 15-min of the final extinction session the day prior (inset; ^$^p = 0.01 by Student’s t-test). (b) Western blot showing total cofilin (T) and S-glutathionylated cofilin (-SSG) in nucleus accumbens extracts from yoked saline (Sal), extinguished (Ext) and reinstated (Rst) animals with quantification below. Levels of S-glutathionylated cofilin are significantly increased during 15-min of cued reinstatement (F _(2, 32)_ = 12.66, p < 0.001; p > 0.999, Saline vs. Extinction; ^$^p = 0.001, Extinction vs. Reinstatement; ^#^p < 0.001, Saline vs. Reinstatement by one-way ANOVA followed by Tukey’s multiple comparisons test; n is shown in legend), although levels of cofilin were unchanged (p = 0.110 by one-way ANOVA). Cofilin-SSG was significantly elevated when normalized to total cofilin from the same animal (F _(2, 32)_ = 0.99, p = 0.004; p = 0.355, Saline vs. Extinction; p = 0.215, Extinction vs. Reinstatement; ^#^p = 0.003, Saline vs. Reinstatement by one-way ANOVA followed by Tukey’s multiple comparisons test). See S2 Fig for full Western blots. (c) Relationship between levels of S-glutathionylated cofilin and total cofilin in nucleus accumbens extracts from yoked saline (blue), cocaine extinguished (yellow) and cue-reinstated (brown) rats. In reinstated, but not extinguished or yoked saline animals, increased levels of cofilin S-glutathionylation correlate significantly with levels of total cofilin (p = 0.327, r^2^ = 0.087, Saline; p = 0.941, r^2^ = 0.001, Extinction; p = 0.001, r^2^ = 0.635, Reinstatement) indicating a protective effect of the modification. (d) Active lever presses during a 15-minute cued reinstatement session correlate positively with levels of cofilin-SSG (p = 0.019, r^2^ = 0.381). Similar levels in high pressers indicate a potential ceiling effect on cofilin S-glutathionylation. Data are presented as mean ± s.e.m. in (a, b).

A targeted proteomic approach to directly measure the extent of site-specific S-glutathionylated cofilin immunoprecipitated from brain tissue only yielded ~45% sequence coverage of the protein without any post-translational modifications (not shown). This, combined with the lack of specific antibodies for S-glutathionylated protein forms, impeded our ability to readily investigate how this post-translational modification is physiologically or pathologically regulated. Thus, to examine cofilin S-glutathionylation in the context of cocaine self-administration and relapse in a rat model, we adapted a biotin switch protocol [21]. We used this assay to detect shifts in levels of S-glutathionylation of cofilin, a protein that has been linked to cocaine-induced changes in excitatory synapse morphology in the nucleus accumbens [32–34]. Rats were trained to self-administer cocaine (Fig 3A) and drug infusion was coupled with a tone/light cue. A control group was yoked to cocaine rats and received saline infusions paired with cues. After extinction from cocaine self-administration without drug or cue exposure (Fig 3A), the conditioned cue was restored to active lever pressing and behavior was reinstated for 15 minutes. Using the S-glutathionylation switch assay, we found that cocaine-associated cues increased cofilin-SSG in tissue extracts from the nucleus accumbens (Fig 3B), a major hub of the reward circuitry in the brain [35]. Although levels of cofilin were not changed between groups, we observed a significant correlation between levels of total cofilin and cofilin-SSG in reinstated, but not in extinguished or control yoked saline animals (Fig 3C) and increased levels of cofilin-SSG were positively correlated with higher levels of active lever pressing in reinstated animals during cue exposure (Fig 3D). Together, these data suggest a protective effect of the GSH modification on cofilin protein levels in drug seeking animals and potentially reduced cofilin turnover compared to extinguished and yoked saline rats.

## Discussion

Chronic use of addictive drugs induces redox stress and produces widespread increases in protein S-glutathionylation [36]. After cocaine administration, cytoplasmic dopamine is rapidly auto-oxidized to superoxide radicals, hydrogen peroxide, and dopamine quinones, which can deplete cellular GSH [37]. Oxidative stress produced by single or repeated daily cocaine injections in rats leads to an increase in total protein S-glutathionylation that persists after weeks of drug withdrawal [36]. The increase corresponds to an increase in the expression of glutathione-S-transferase P (GSTP), the enzyme that catalyzes the addition of GSH to target cysteines, suggesting that cocaine induces long-lasting adaptations to the glutathione system. Moreover, GSTP1 polymorphisms have been linked to human psychostimulant addiction in two distinct populations [38, 39], and inhibiting GSTP in rodents augments locomotion in a behavioral sensitization model of cocaine-induced neuronal plasticity [36].

Although cofilin-SSG was not significantly elevated after extinction from cocaine self-administration as we expected based on previous results [36], 15-minutes of cued reinstatement in the absence of cocaine infusions initiated increases in cofilin-SSG relative to yoked saline controls, as well as tissue from extinguished animals that also underwent cocaine self-administration (Fig 3B). Since all rats were habituated to the operant chamber as well as experimenter handling over 20 d of training, we do not expect the operant chamber or home cage to induce differential effects on cofilin glutathionylation, particularly in yoked saline animals. However, exposure to the extinguished context has been shown to induce post-synaptic adaptations, including AMPA receptor insertion, in the nucleus accumbens in a cell type-specific manner [40]. A limitation of this study is that tissue was extracted from yoked controls and extinguished rats 24h after the last exposure to the operant chamber, while tissue was extracted from reinstated animals after 15 min in the operant chamber. Thus, our experimental design does not directly distinguish between an induction of cofilin-SSG arising from 15 min in the extinguished context or from 15 min of cued reinstatement. However, we did not detect differences 24h after the last extinction session, and the positive correlation between cofilin-SSG and active lever pressing supports an involvement of cue responding. Whether acute oxidative stress from dopamine release in response to the cues previously paired with cocaine [41] initiated the increase in cofilin-SSG or whether this change was mediated by chronic cocaine-induced adaptations in the cell signaling that directs post-translational S-glutathionylation is an important question. Signaling by nNOS interneurons during cued cocaine seeking [42] may also play a role in driving redox shifts that lead to increased cofilin-SSG, since superoxide radicals readily react with NO to form peroxynitrite, a reactive nitrogen species. Furthermore, cocaine is known to decrease astrocyte expression of the cystine-glutamate antiporter, system xc- [43], and downregulation of this antiporter would limit availability of intracellular cysteine for GSH production in astrocytes. Since neurons are dependent on astrocytes for redox buffering [44], such astrocytic changes could contribute to reduced redox flexibility in neurons after chronic cocaine use.

Synaptic plasticity depends on actin dynamics in dendritic spines [45] and spines in the nucleus accumbens exhibit dynamic morphological changes, including spine head expansion, after 15-min of exposure to cocaine-conditioned cues that reliably induce drug seeking behavior [46]. Thus, S-glutathionylation-induced inhibition of cofilin could contribute to cocaine cue-induced synaptic plasticity, which depends on morphological adaptations in dendritic spines, and associated drug-seeking behavior. Dendritic spine structure is inherently linked to synaptic function [10], and the spine cytoskeleton is formed largely by F-actin, which provides the structural framework for receptor trafficking and synaptic plasticity-associated alterations in spine morphology [47]. The existence of multiple post-translational modifications on cofilin (e.g. S-glutathionylation and phosphorylation) to shape dendritic spine morphology is in keeping with the rapid timescale of plasticity-associated changes in spine dynamics. Furthermore, the ability of various antioxidants to interfere with cocaine self-administration and cue-induced cocaine seeking is consistent with the idea that the compulsive reinforcing actions of cocaine and vulnerability to relapse result in part from effects on cellular redox [48–50].

Based on the findings in this report, we hypothesize that S-glutathionylation of cofilin is a modification that may contribute to changes in dendrite morphology during cue-induced seeking. The S-glutathionylation of structural filament proteins and related cofactors, such as cofilin, may constitute a mechanism for regulating cell morphology based on changes in local in redox status, such as that which occurs during dopamine release in response to drug reward. Thus, our findings are consistent with the possibility that S-glutathionylation-induced inactivation of cofilin can be a mechanism for cells within the nucleus accumbens to translate local dopamine levels to morphological changes that affect synaptic plasticity.

## Supporting information

S1 Table S-glutathionylated peptides and diagnostic fragment ions of human recombinant cofilin 1 observed by LC-MS/MS using different fragmentation modes. Depending on the sequence and precursor ion charge state, S-glutathionylated peptides may undergo neutral losses of 129 Th following collisional dissociation [51, 52] or 305 Th following ETD [53] which can complicate automated detection or be used as a diagnostic filter. To evaluate instrument parameters and fragmentation modes enabling direct detection of S-glutathionylated peptides, LysC digested cofilin-1 was analyzed by LC-MS/MS using CID-induced neutral loss-triggered acquisition of ETD spectra, HCD and ETD with supplemental activation enabled (ETD+SA) alternating, or acquisition of alternating CID and ETD spectra. By CID, the most abundant ion in the spectra resulted from neutral loss of 129 (glutamic acid from glutathione) from the precursor. For doubly and triply charged precursor ions, this neutral loss could be used to trigger acquisition of a complementary ETD spectrum on the same precursor. In contrast, by HCD neutral loss of 129 from the precursor ion was not observed or was of low abundance and product ions underwent partial neutral loss of 129 Th. ETD MS/MS results in dissociation at the disulfide bond yielding abundant neutral loss of 305 Th from the precursor ion and a diagnostic glutathione oxonium ion 3. Detection of the diagnostic ion depended on the charge state as not all 2+ ions yielded the glutathione ion at m/z 308. ETD+SA yielded more complex spectra with neutral losses of both 129 and 305 Th. Given the sensitivity and faster scan rate in CID compared to HCD, cofilin immunoprecipitated from nucleus accumbens was analyzed using CID with an inclusion list (m/z indicated by asterisk). Missed cleavage (MC); collision activated dissociation (CID); higher-energy C-trap dissociation unique to Orbitrap mass spectrometers (HCD); electron transfer dissociation (ETD); tandem mass spectrometry (MS/MS).
(TIF)

S2 Table Database search algorithm scores for S-glutathionylated peptides of cofilin following MS/MS fragmentation by CID, higher-energy collisional dissociation (HCD), or ETD with or without supplemental activation (SA) enabled. LC-MS/MS analyses were performed with alternating CID/ETD, HCD/ETD+SA, or neutral loss (129 Th) triggered acquisition of ETD MS/MS. HCD and CID spectra were searched with possible neutral loss (NL) of 129 Da. ETD spectra were searched with possible diagnostic glutathione ion at 308 m/z. Peptide fragments generated by CID and ETD were detected in the ion trap. HCD spectra were mass analyzed in the Orbitrap. Scores and thresholds are specific to each algorithm. MaxQuant and Protein Prospector yielded scores for each peptide above those typically used for filtering out false positives. These data are consistent with a previous study that found the Mascot database searching algorithm identified S-glutathionylated peptides fragmented by HCD more consistently than those fragmented by CID or ETD [54].
(TIF)

S1 Fig Mass spectra confirming cofilin residues capable of undergoing S-glutathionylation. Cofilin 1 peptides were analyzed by ETD, CID, and HCD mass spectrometry and all four cysteines in the protein (a, C39; b, C80; c, C139; and d, C147) were found to undergo S-glutathionylation *in vitro*.
(JPG)

S2 Fig Full Western blots showing total cofilin and cofilin-SSG extracted from the nucleus accumbens of animals that underwent cocaine self-administration. Numbers on the left indicate experimental replicates. Cofilin and cofilin-SSG were detected ~20 kDa using a polyclonal antibody (ab42824). All blots show total cofilin (T) and cofilin-SSG (G) side by side for each animal. Behavioral groups are indicated above blots (yoked saline, S; extinction, E; reinstatement, R). Western blot lanes showing undetectable or abnormal/uneven signal indicative of improper protein transfer were excluded from analyses (indicated by X). A portion of samples in experimental replicates 1 and 3 included conditions not relevant to this study (indicated by—).
(TIF)
