# Supplementary figures and images for: Post-translational S-glutathionylation of cofilin increases actin cycling during cocaine seeking
Source: PLoS One. 2019 Sep 24;14(9):e0223037. doi: 10.1371/journal.pone.0223037 (PMC6759170; doi:10.1371/journal.pone.0223037)

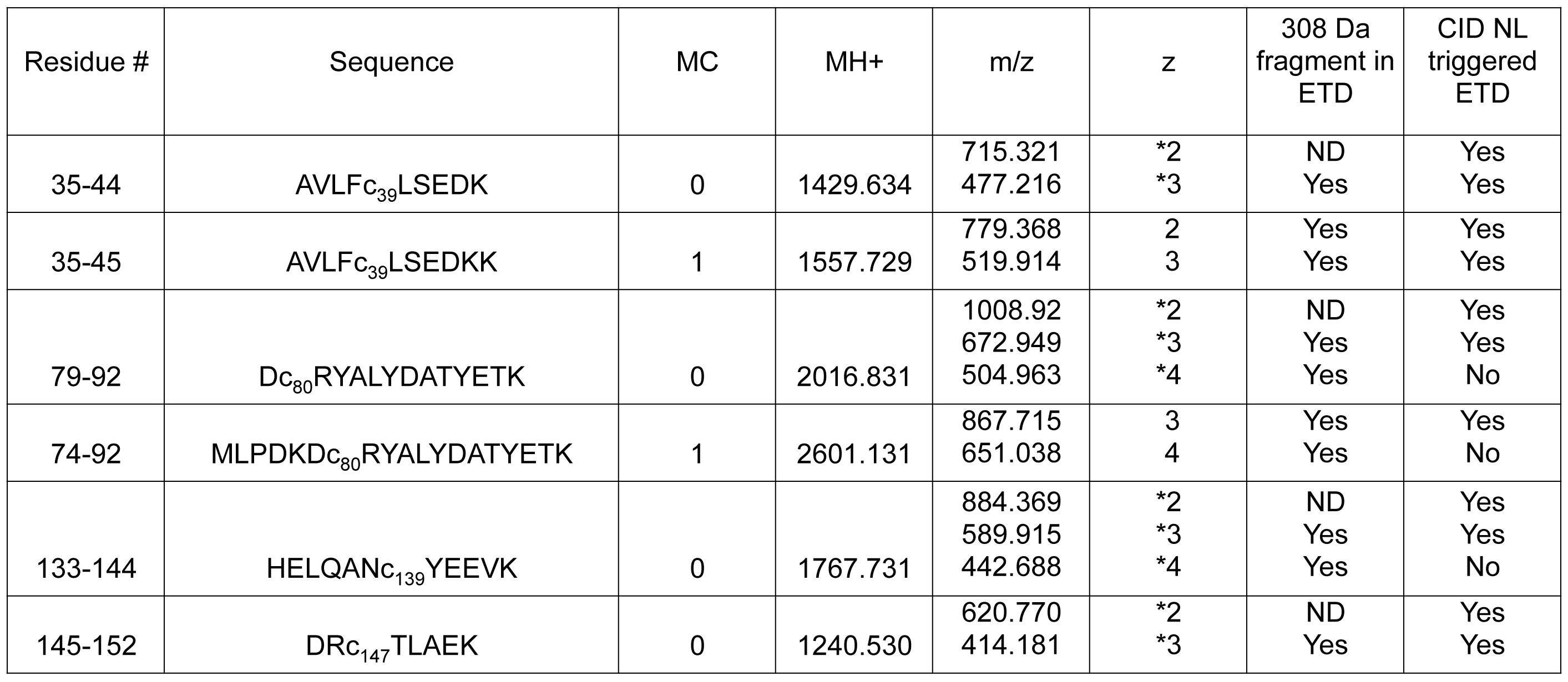

Supplement: S1 Table — Depending on the sequence and precursor ion charge state, S-glutathionylated peptides may undergo neutral losses of 129 Th following collisional dissociation [51, 52] or 305 Th following ETD [53] which can complicate automated detection or be used as a diagnostic filter. To evaluate instrument parameters and fragmentation modes enabling direct detection of S-glutathionylated peptides, LysC digested cofilin-1 was analyzed by LC-MS/MS using CID-induced neutral loss-triggered acquisition of ETD spectra, HCD and ETD with supplemental activation enabled (ETD+SA) alternating, or acquisition of alternating CID and ETD spectra. By CID, the most abundant ion in the spectra resulted from neutral loss of 129 (glutamic acid from glutathione) from the precursor. For doubly and triply charged precursor ions, this neutral loss could be used to trigger acquisition of a complementary ETD spectrum on the same precursor. In contrast, by HCD neutral loss of 129 from the precursor ion was not observed or was of low abundance and product ions underwent partial neutral loss of 129 Th. ETD MS/MS results in dissociation at the disulfide bond yielding abundant neutral loss of 305 Th from the precursor ion and a diagnostic glutathione oxonium ion 3. Detection of the diagnostic ion depended on the charge state as not all 2+ ions yielded the glutathione ion at m/z 308. ETD+SA yielded more complex spectra with neutral losses of both 129 and 305 Th. Given the sensitivity and faster scan rate in CID compared to HCD, cofilin immunoprecipitated from nucleus accumbens was analyzed using CID with an inclusion list (m/z indicated by asterisk). Missed cleavage (MC); collision activated dissociation (CID); higher-energy C-trap dissociation unique to Orbitrap mass spectrometers (HCD); electron transfer dissociation (ETD); tandem mass spectrometry (MS/MS). (TIF) [file pone.0223037.s001.tif]

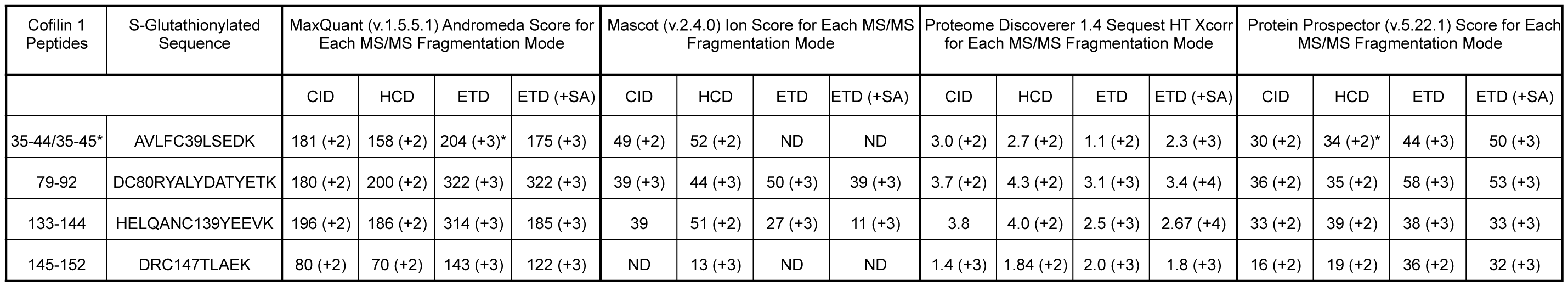

Supplement: S2 Table — LC-MS/MS analyses were performed with alternating CID/ETD, HCD/ETD+SA, or neutral loss (129 Th) triggered acquisition of ETD MS/MS. HCD and CID spectra were searched with possible neutral loss (NL) of 129 Da. ETD spectra were searched with possible diagnostic glutathione ion at 308 m/z. Peptide fragments generated by CID and ETD were detected in the ion trap. HCD spectra were mass analyzed in the Orbitrap. Scores and thresholds are specific to each algorithm. MaxQuant and Protein Prospector yielded scores for each peptide above those typically used for filtering out false positives. These data are consistent with a previous study that found the Mascot database searching algorithm identified S-glutathionylated peptides fragmented by HCD more consistently than those fragmented by CID or ETD [54]. (TIF) [file pone.0223037.s002.tif]

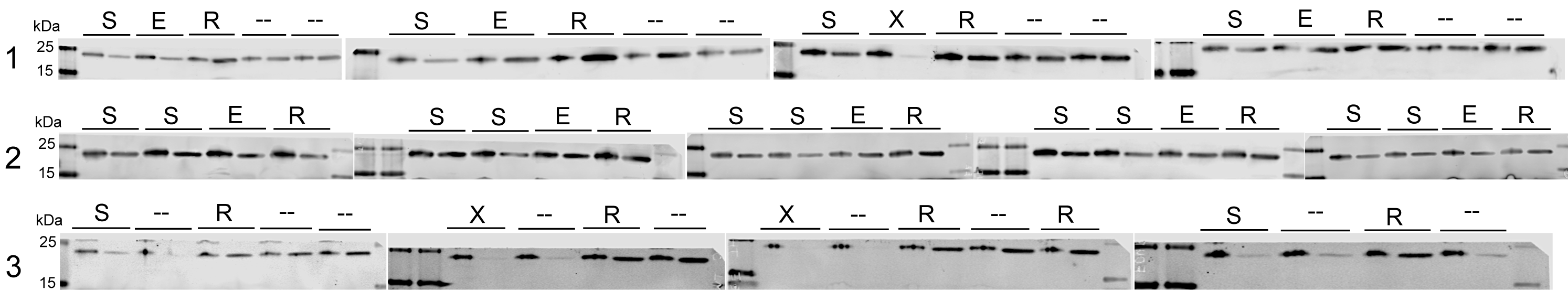

Supplement: S2 Fig — Numbers on the left indicate experimental replicates. Cofilin and cofilin-SSG were detected ~20 kDa using a polyclonal antibody (ab42824). All blots show total cofilin (T) and cofilin-SSG (G) side by side for each animal. Behavioral groups are indicated above blots (yoked saline, S; extinction, E; reinstatement, R). Western blot lanes showing undetectable or abnormal/uneven signal indicative of improper protein transfer were excluded from analyses (indicated by X). A portion of samples in experimental replicates 1 and 3 included conditions not relevant to this study (indicated by—). (TIF) [file pone.0223037.s004.tif]
